# Consequences of screening in abdominal aortic aneurysm: development and dimensionality of a questionnaire

**DOI:** 10.1186/s41687-018-0066-1

**Published:** 2018-09-04

**Authors:** John Brodersen, Anders Hansson, Minna Johansson, Volkert Siersma, Marcus Langenskiöld, Monica Pettersson

**Affiliations:** 1 Section and Research Unit for General Practice, Department of Public Health, Faculty of Health Sciences, University of Copenhagen, Øster Farimagsgade 5, P. O. Box 2099, DK-1014 Copenhagen, Denmark; 2 Primary Health Care Research Unit, Sorø, Zealand Region Denmark; 3 The University Health Care Research Center, Region Örebro County, School of Medical Sciences, Örebro University, Örebro, Sweden; 4 Research Unit and Section for General Practice, FoUU-centrum Fyrbodal, 46235 Vänersborg, Sweden; 5 Department of Public Health and Community Medicine, Institute of Medicine, Sahlgrenska Academy, University of Gothenburg, Gothenburg, Sweden; 6 Department of Vascular Surgery and Institute of Medicine, Department of Molecular and Clinical Medicine, Sahlgrenska University Hospital and The Sahlgrenska Academy at the University of Gothenburg, Gothenburg, Sweden; 7 Department of Vascular Surgery, Sahlgrenska University Hospital, and Sahlgrenska Academy, University of Gothenburg, Institute of Health and Care Sciences, Gothenburg, Sweden

## Abstract

**Background:**

In interview studies, men under surveillance for screening-detected abdominal aortic aneurysms have reported ambivalence towards this diagnosis: the knowledge was welcomed together with worries, feelings of anxiety and existential thoughts about life’s fragility and mortality due to the diagnosis. Previous surveys about health-related quality of life aspects among men under surveillance for screening-detected aneurysm have all used generic patient-reported outcomes. Therefore, the aim of this study was to extend the core-questionnaire Consequences of Screening for use in abdominal aortic aneurysm screening by testing for comprehension, content coverage, dimensionality, and reliability.

**Methods:**

In interviews, the suitability, content coverage, and relevance of the core-questionnaire Consequences of Screening were tested on men under surveillance for a screeningdetected abdominal aortic aneurysm. The results were thematically analysed to identify the key consequences of abnormal screening results. Item Response Theory and Classical Test Theory were used to analyse data. Dimensionality, differential item functioning, local response dependency and reliability were established by item analysis, examining the fit between item responses and Rasch models.

**Results:**

The core-questionnaire Consequences of Screening was found to be relevant for men offered regular follow-up of an asymptomatic screening-detected abdominal aortic aneurysm.

Fourteen themes especially relevant for men diagnosed with a screening-detected abdominal aortic aneurysm were extracted from the interviews: ‘Uncertainty about the result of the ultra sound examination’, ‘Change in body perception’, ‘Guilt’, ‘Fear and powerlessness’, ‘Negative experiences from the examination’, ‘Emotional reactions’, ‘Change in lifestyle’, ‘Better not knowing’, ‘Fear of rupture’, ‘Sexuality’, ‘Information’, ‘Stigmatised’, ‘Self-blame for smoking’, ‘Still regretful smoking’. Altogether, 55 new items were generated: 3 were single items and 13 were only relevant for former or current smokers. 51 of the 52 items belonging to a theme were confirmed to fit Rasch models measuring fourteen different constructs. No differential item functioning and only minor local dependency was revealed between some of the 51 items.

**Conclusions:**

The reliability and the dimensionality of a condition-specific measure with high content validity for men under surveillance for a screening-detected abdominal aortic aneurysm have been demonstrated. This new questionnaire called COS-AAA covers in two parts the psychosocial experience in abdominal aortic aneurysm screening.

## Background

Abdominal aortic aneurysm (AAA) is a life-threatening condition that may lead to death due to sudden rupture of the aorta. Risk factors for developing AAA are smoking, male sex, advanced age and family history [1]. Therefore, prevention of AAA and its complications is done by smoking cessation (primary prevention) and early detection via screening (secondary prevention). Screening for AAA reduces AAA-related mortality by reducing the number of AAA-ruptures [2] and has therefore been introduced in Sweden, the US and the UK during the last two decades [3]. Approximately 50% of screening-detected AAA will in 5 years reach an aortic diameter for which surgery is recommended [4]. It is suggested that approximately 45% of men with screening-detected AAAs are overdiagnosed because their aneurysm would never have led to symptoms or death [2, 3]. These men have to live with the fear of a life-threatening condition and are offered regular ultrasound surveillance throughout their remaining life [5]. No condition-specific questionnaire to measure psychosocial aspects or quality of life of participants in AAA screening is available. The development and validation of such an instrument would be of great importance in evaluating the balance between the potential benefits and the potential harms of AAA screening. Moreover, such an instrument could also potentially lead to an improvement of the care and information given to men attending aortic surveillance programmes.

In interview studies, men under surveillance for screening-detected AAAs have reported ambivalence towards the knowledge of having an AAA and towards lines of actions because of the condition. The knowledge was welcomed together with worries, feelings of anxiety and existential thoughts about life’s fragility and mortality due to the diagnosis. These men experienced anxiety about the risk of rupture [6–10]. However, because these studies are qualitative they cannot estimate the degree or extent of psychosocial harms. We have identified six quantitative studies about psychological aspects and quality of life (QoL) following the diagnosis of a screening-detected AAA [11–16]. One study displayed decreases in QoL 12 months after screening [15]. The other five studies indicated no clinically important decrease in QoL compared to unscreened men [11–14, 16]. However, each of these studies used generic questionnaires to measure the psychological aspects and QoL, e.g. SF-36, ScreenQL, EQ-5D and HADS [11–16]. Generic questionnaires may have lack of content validity compared to condition-specific questionnaires [17, 18]. This means that aspects that might be specifically important for men with screening-detected AAAs are lacking, e.g. anxiety about rupture during sexual activity. Additionally, aspects irrelevant for this specific group can lead to incorrect inferences [17, 18]. Therefore, the use of generic instruments is questioned in a screening context [19–21]. The lack of studies using condition-specific questionnaires is pointed out in two recent systematic reviews [1, 22].

Three condition-specific questionnaires with high content validity and adequate psychometric properties (using Rasch modelling) have previously been developed by Brodersen et al., to measure short and long term psychosocial consequences in breast cancer screening (the Consequence of Screening in Breast Cancer (COS-BC)) [23, 24], in lung cancer screening (Consequence of Screening in Lung Cancer (COS-LC)) [25] and in cervical cancer screening (Consequence of Screening in Cervical Cancer (COS-CC)) [26]. In the qualitative studies conducted when developing these three measures a common core-questionnaire COS was revealed. Moreover, some of the informants perceived the cancer they were screened for as a non-communicable life threatening disease [23, 25, 26]. Also the men who took part in our previously conducted qualitative study had a perception of being under surveillance for a non-communicable life threatening disease, e.g. by some of the men described as “a ticking bomb inside your stomach” [7]. An unanswered question is if COS is also relevant in a setting of AAA screening. Therefore, the aim of the present study was threefold:

1. to examine the content relevance and content coverage of the core items of the COS in a setting of AAA screening among men under surveillance for a screening-detected AAA;
2. if lack of content coverage of the COS was revealed, to generate themes and new items especially relevant for men under surveillance for a screening-detected AAA and to test the items for suitability;
3. if new items were generated, to test the extended version of the COS for dimensionality using Item Response Theory Rasch models.

## Methods

### Data collection: Interviews about content relevance and content coverage of the COS for application in AAA screening

Fifteen men with screening-detected AAA were recruited in 2010 for single interviews [7]. In the present study, these men were re-invited in groups of five to participate in three group interviews that took place on August and September 2012. From 2010 to 2012 all 15 men had had at least one follow-up ultrasound examination. The group interviews took place in a non-hospital setting.

Before the first group interview, the transcriptions of the previously conducted 15 qualitative interviews were re-read [7] and compared with the subject matter of items in the COS-BC, the COS-LC and the COS-CC [23, 25, 26]. These potentially AAA-screening-specific items were thereafter translated from Danish into Swedish and checked by a bilingual person who had Danish as mother tongue. A validated Swedish version of the COS has been published and was used in this study [27]. The potential AAA-screening-specific items together with the COS items were tested in the first group interview for relevance. If the potential AAA-screening-specific items were found relevant they were thereafter regarded as ‘new’ items in an AAA context.

The group interviews consisted of two parts: first, an open-ended discussion on the psychosocial consequences of being diagnosed with an AAA via screening. The conceptualisation of ‘psychosocial consequences’ was based on Engel’s the bio-psycho-social model [28]. Second, the participants were asked to complete a draft of a questionnaire encompassing: the COS, the potentially AAA-screening-specific items plus any newly generated items from the previously open-ended discussion(s). After completing the draft of the questionnaire the group participants were asked to discuss if these items had been – or were – relevant for them at any time during the period from their first screening visit until now.

COS consists of two parts. Part I of the COS encompasses one single item and four dimensions, in total 25 items [23–25]. If some of the potentially AAA-screening-specific items were found relevant and if new items were generated in the open-ended discussions, the participants in the following group interviews would be asked to complete a draft to a new questionnaire called COS-AAA (Consequences Of Screening in Abdominal Aortic Aneurysm) encompassing: relevant items from the COS plus items identified in the preceding group interviews. Part II of the COS encompasses six dimensions including 23 items [23, 25]. The theme “breast/lung/cervical cancer” encompassing two items in Part II was for obvious reasons deleted from the part II of the COS. The COS-items are ordered thematically in Table 1.

**Table 1:**
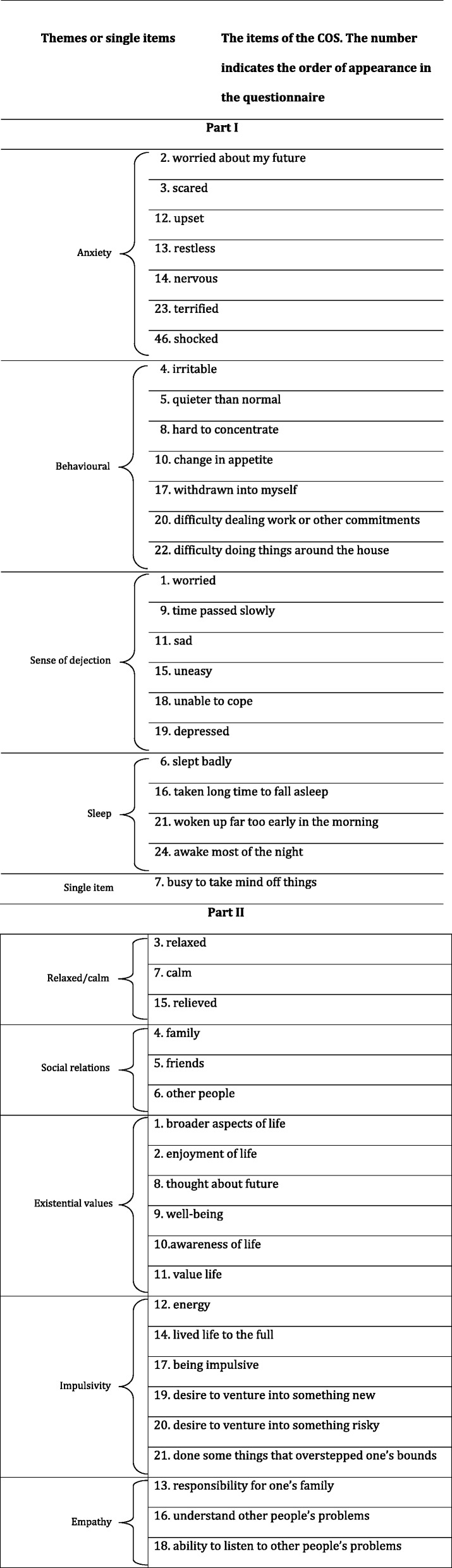
Content of the core-questionnaire COS (Consequence of Screening)

In the group interviews, cognitive interviewing was also carried out item-by-item and covered understandability and content coverage [29, 30].

**Fig. 1:**
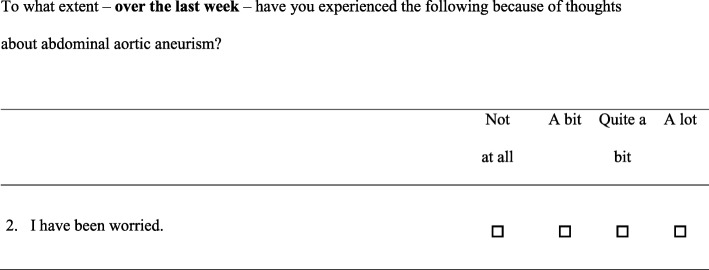
The layout and response categories for part I of the Consequences of Screening questionnaire

**Fig. 2:**
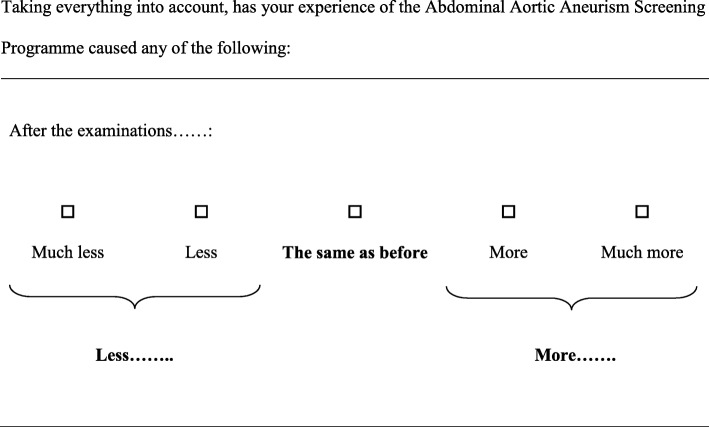
The layout and response categories for part II of the Consequences of Screening questionnaire

The response options were also reviewed for relevance and ease of completion. In part I there are four ordinal categorical response options: ‘Not at all’, ‘A bit’, ‘Quite a bit’ and ‘A lot’ (Fig. 1). The five response options in part II: ‘Much less’, ‘Less’, ‘The same as before’, ‘More’ and ‘Much more’ are also ordinal categorical variables that are partially ordered (Fig. 2).

The COS-BC part II was developed so that each item included response options indicating ‘no change’ as an anchor relative to two other options of changes in opposing directions. It follows that any change from ‘The same as before’ is to be regarded as a long-term psychosocial consequence of an abnormal screening result [25, 31]. Therefore, the responses to part II should be recoded: a response to ‘Much less’ or ‘Much more’ becomes a response to one variable of ‘much less/more change’, a response to ‘Less’ or ‘More’ becomes a response to one variable of ‘less/more change’ and finally a response to ‘The same as before’ becomes a response to a variable of ‘no change’ [25, 31].

The group interviews were audio-recorded. After each interview the recording was independently audited by two authors conducting thematic analyses to determine the key psychosocial consequences. In the subsequent group interviews the identified themes were discussed. Furthermore, the participants’ verbatim comments were used to define a construct, e.g. *negative experiences from the examination*.

### Data collection for statistical psychometric analyses

The draft of the COS-AAA was posted from January to April 2013 to 250 men who had been diagnosed with an AAA via screening and 500 men who had received a normal screening result. Eligible were men who had participated in Västra Götaland’s AAA screening programme aged 65 years. The men were asked to complete the questionnaire and return it in an enclosed stamped addressed envelope.

### Psychometric statistical analyses

To provide measurement of psychosocial consequences consistent with Rasch philosophy, the scales calculated from the data collected for psychometric analysis should fit a partial credit Rasch model [32]. If a scale did not fit this model, the data were used to identify particular problems and to give directions how to adjust the scales so as to repair these.

Overall assessment of a scale’s homogeneity and differential item functioning (DIF) was evaluated by Andersen’s conditional likelihood ratio test [33]. Homogeneity was tested by comparing the two subgroups in the data defined by a dichotomisation of the total score on all items. DIF was tested by comparing the subgroups defined by the categories of specific exogenous variables: screening result, social group, education level, income and mother tongue. Local response dependency (LD) was identified using graphical log-linear Rasch models (GLLRM) [34].

If the overall tests indicated problems with the Rasch model fit of a scale, the individual items were investigated; GLLRMs were employed here where specific tests for conditional independence identify particular problems [34]. Individual item attribution to the heterogeneity of the scale was assessed by conditional infits and outfits. Infits are chi-square statistics with each observation weighted by its statistical information; they are sensitive to patterns of responses by persons on items that are targeted on them. Outfits are conventional chi-square statistics; they are sensitive to responses by persons on items that are very easy or very hard for them [35]. By analysis of the association between items and their *rest-scores,* i.e. the total score with the corresponding item removed; an item shows misfit if this association is different from the association expected in a Rasch model [34, 35]. DIF in individual items relative to the aforementioned exogenous variables was assessed by a test for the association between the items and the exogenous variables adjusted for the total score [36]. Likewise, LD was assessed by the association between item pairs adjusted for the appropriate rest-score [34, 37]. In these accounts, the Benjamini-Hochberg procedure was used to account for multiple testing [38]. Items exhibiting the most problematic behaviour relative to the above tests were deleted from the scales sequentially until the scale fitted the Rasch model, e.g. the functionality of the item’s response categories [25].

Reliability was assessed by Cronbach’s alpha [39, 40].

All analyses were carried out using DIGRAM [41].

The single items were not included in the Rasch analyses because these items did not belong to any theme.

The study was partly funded by FoUU-centrum Fyrbodal. The study was approved by the Regional Ethical Review Board in Gothenburg. Informed consent was obtained from all individual participants included in the study.

## Results

### Results from the interviews

Five (100%), four (80%) and two (40%) men accepted each to participate in group interviews.

The items in the COS were all found relevant by the participants. Fourteen themes especially relevant for men diagnosed with a screening-detected AAA were extracted from the interviews: ‘Uncertainty about the result of the ultra sound examination’, ‘Change in body perception’, ‘Guilt’, ‘Fear and powerlessness’, ‘Negative experiences from the examination’, ‘Emotional reactions’, ‘Change in lifestyle’, ‘Better not knowing’, ‘Fear of rupture’, ‘Sexuality’, ‘Information’, ‘Stigmatised’, ‘Self-blame for smoking’, ‘Still regretful smoking’ (Table 2). The latter three themes: ‘Stigmatised’, ‘Self-blame for smoking’, ‘Still regretful smoking’ were only relevant for former or current smokers. Altogether, 55 AAA-screening-specific items for part I were generated of which 3 were single items and 13 were only relevant for former or current smokers (Tables 3 and 4). The 14 themes and the subject matter for all 55 AAA-screening-specific items were generated in the first group interview and accepted in the following group interviews. The response options were found relevant, comprehensive and easy to complete.

**Table 2:** Fit statistics and reliability (Cronbach’s alpha) of the dimensions of the COS-AAA

Dimensions (Number of items)	CLR-χ^2^	Degrees of freedom	P	Cronbach’s alpha
Anxiety (7)	21.71	16	0.1528	0.789
Behavioural (7)	24.54	19	0.1761	0.804
Behavioural (6) minus item 9	10.00	16	0,8667	0.804
Sense of dejection (6)	37.24	16	0.0019^d^	0.830
Sense of dejection (6)^c^	24.24	26	0.5622	0.830
Sleep (4)	51.19	11	< 0.0001^d^	0.807
Sleep (3), minus item 23	6.55	8	0.5853	0.840
Uncertainty about the result of the ultra sound examination (3)^b^	10.13	7	0.1812	0.343
Change in body perception (8)	21.45	19	0.3124	0.838
Guilt (2)^b^	0.00	4	1.0000	0.594
Fear and powerlessness (7)	6.30	19	0.9971	0.849
Negative experiences from the examination (2)	4.93	4	0.2948	0.592
Emotional reactions (3)	2.38	6	0.8820	0.790
Change in lifestyle (2)	0	5	1.0000	0.600
Better not knowing (2)^b^	1.22	4	0.8753	0.704
Fear of rupture (4)	7.08	9	0.6286	0.779
Sexuality (3)^b^	1.8	8	0.9865	0.820
Information (3)^b^	24.77	8	0.0017^d^	0.595
Information (2), minus item 65	0	3	1.0000	0.937
Stigmatised (4)^a^	5.23	11	0.9193	0.873
Self-blame for smoking (3)^a^	5.24	8	0.7312	0.914
Still regretful smoking (6)^a^	4.92	5	0.4262	0.890
Existential values (6)	24.66	10	0.0061^d^	0.832
Relaxed/calm (3), item 3 possess DIF in relation to diagnosis	3.11	5	0.6831	0.727
Relaxed/calm (2), minus item 3	1.65	3	0.6474	0.716
Social network (3)	8.80	4	0.0662	0.766
Impulsivity (6)	4.83	11	0.9389	0.830
Empathy (3)	6.31	5	0.2776	0.619

^a^Scales only relevant to former and current smokers

^b^Only for those men diagnosed with screening-detected AAA

^c^Graphical log-linear Rasch model where two super items were included: item 1 & item 9 and item 1 & item 15

^d^Misfit after a correction by the Benjamini-Hochberg procedure [38]

**Table 3:** Summary of result from the psychometric analyses of part 1 of the COS-AAA

The items of part I of the COS-AAA in order of the scales (the item number indicates the order of appearance in the questionnaire)	Subscales and misfit	Observed	Expected	Gamma sd	Probability of fit to the Rasch model	Single or ‘poor’ item
2. worried about my future	Anxiety	0.754	0.757	0.039	0.94248	
3. scared	Anxiety	0.879	0.776	0.055	0.06203	
11. upset	Anxiety	0.823	0.768	0.046	0.23547	
12. restless	Anxiety	0.652	0.753	0.041	0.01299^a^	
13. nervous	Anxiety	0.834	0.762	0.045	0.10958	
22. terrified	Anxiety	0.819	0.786	0.078	0.67539	
46. shocked	Anxiety	0.635	0.777	0.063	0.02409^a^	
4. irritable	Behavioural	0.702	0.728	0.040	0.51894	
5. quieter than normal	Behavioural	0.681	0.728	0.041	0.25644	
7. hard to concentrate	Behavioural	0.805	0.727	0.040	0.05137	
9. change in appetite	Behavioural	0.535	0.746	0.053	0.00006^b^	
16. withdrawn into myself	Behavioural	0.812	0.728	0.050	0.09095	
20. difficulty dealing work or other commitments	Behavioural	0.818	0.760	0.059	0.32877	
21. difficulty doing things around the house	Behavioural	0.775	0.736	0.047	0.40838	
1. worried	Dejection	0.870	0.860	0.025	0.69871	
8. time passed slowly	Dejection	0.582	0.676	0.059	0.11251	
10. sad	Dejection	0.893	0.820	0.035	0.03861	
14. uneasy	Dejection	0.878	0.891	0.021	0.55835	
17. unable to cope	Dejection	0.769	0.819	0.034	0.13788	
18. depressed	Dejection	0.901	0.819	0.039	0.03908^a^	
6. slept badly	Sleep	0.907	0.872	0.022	0.11844	
15. taken long time to fall asleep	Sleep	0.839	0.858	0.027	0.46644	
24. awake most of the night	Sleep	0.870	0.884	0.023	0.52241	
32. surprised something was wrong (3-item scale)	Uncertainty about the results of the ultra sound examination	0.182	0.368	0.101	0.06646	
39. uncertain about the actual meaning of the examination result (3-item scale)	Uncertainty about the results of the ultra sound examination	0.474	0.352	0.107	0.25358	
39. uncertain about the actual meaning of the examination result (2-item scale)	Uncertainty about the results of the ultra sound examination	0.942	0.942	0.023	0.99604	
40. difficulties in accepting that the examination result was correct (3-item scale)	Uncertainty about the results of the ultra sound examination	0.497	0.331	0.143	0.24422	
40. difficulties in accepting that the examination result was correct (2-item scale)	Uncertainty about the results of the ultra sound examination	0.942	0.942	0.023	0.99604	
31. different sense in the body	Change in body perception	0.739	0.765	0.038	0.48972	
38. thought my body was vulnerable	Change in body perception	0.830	0.766	0.040	0.11518	
42. felt older than my age	Change in body perception	0.828	0.773	0.044	0.21437	
49. felt that my body was not my own body	Change in body perception	0.858	0.781	0.071	0.27448	
47. feels like something is wrong with my body	Change in body perception	0.857	0.773	0.040	0.03655^a^	
57. felt that I am getting older	Change in body perception	0.677	0.771	0.040	0.01867^a^	
58. noted if I could feel something was different in my stomach	Change in body perception	0.694	0.764	0.039	0.07827	
61. experienced that my body was a machine that does not work	Change in body perception	0.821	0.770	0.042	0.21602	
33. my own fault	Guilt	0.772	0.772	0.092	0.99999	
55. wondered if I should have taken better care of myself	Guilt	0.772	0.772	0.092	0.99999	
26. fragile	Fear and powerlessness	0.731	0.784	0.038	0.15788	
30. felt vulnerable	Fear and powerlessness	0.796	0.796	0.045	0.99903	
37. felt unsafe	Fear and powerlessness	0.843	0.808	0.045	0.43550	
43. felt I was unlucky	Fear and powerlessness	0.735	0.793	0.039	0.13855	
50. the situation seemed hopeless	Fear and powerlessness	0.876	0.811	0.046	0.15857	
54. felt powerless	Fear and powerlessness	0.856	0.801	0.044	0.20922	
60. experienced that I lost control	Fear and powerlessness	0.892	0.834	0.050	0.24566	
29. unpleasant examination(s)	Negative experiences from the examination	0.891	0.891	0.064	0.99928	
48. felt vulnerable at the examination bed	Negative experiences from the examination	0.891	0.891	0.064	0.99928	
25. angry	Emotional reactions	0.912	0.914	0.021	0.94175	
28. felt sour (attitude)	Emotional reactions	0.911	0.885	0.028	0.36390	
56. experienced mood swings	Emotional reactions	0.885	0.902	0.022	0.45158	
36. changed exercise habits	Change in lifestyle	0.767	0.765	0.046	0.96988	
44. changed diet habits	Change in lifestyle	0.767	0.765	0.046	0.96988	
27. regret screening examination	Better not knowing	0.958	0.960	0.031	0.95912	
45. better not to know about the aneurism	Better not knowing	0.958	0.960	0.031	0.95912	
34. fear of rupture at the back of one’s mind	Fear of rupture	0.830	0.818	0.048	0.80348	
35. worried about rupture at hard physical activities	Fear of rupture	0.893	0.801	0.050	0.06566	
52. worried if I had to push myself	Fear of rupture	0.863	0.777	0.094	0.36041	
53. do not dare to push myself as usual	Fear of rupture	0.645	0.781	0.060	0.02442§	
62. less interest in sex	Sexuality	0.897	0.878	0.035	0.57648	
63. negative impact on my sex life	Sexuality	0.899	0.876	0.035	0.50434	
64. worried about rupture of the aneurism during sexual activities	Sexuality	0.803	0.862	0.047	0.20584	
66. searched the Internet for knowledge about aneurism	Information	0.991	0.991	0.006	0.99997	
67. I have looked for knowledge about how the aorta can change	Information	0.991	0.991	0.006	0.99997	
19. busy to take mind off things	Single item	Not included	Not included	Not included	Not included	Single item
23. woken up far too early in the morning	Misfit (Sleep)	0.600	0.770	0.031	< 0.00001^b^	‘Poor’ or single item
41. thought about death	Single item	Not included	Not included	Not included	Not included	‘Poor’ or single item
51. felt sick	Single item	Not included	Not included	Not included	Not included	‘Poor’ or single item
59. more tired than usual	Single item	Not included	Not included	Not included	Not included	‘Poor’ or single item
65. missed information about physical activities and aneurism	Misfit (Information)	0.357	0.593	0.077	0.00199^b^	‘Poor’ or single item

^a^Adjusting the *p*-values in the table in order to control the false discovery rate and so avoid spurious significant results due to multiple testing suggested that this result should be regarded as not significant [38]

^b^Misfit after a correction by the Benjamini-Hochberg procedure [38]

**Table 4:** Summary of result from the psychometric analyses of item to former and current smokers

The items in order of the scales (the item number indicates the order of appearance in the questionnaire)	Subscales and misfit to the Rasch model	Observed	Expected	Gamma sd	Probability of fit to the Rasch model
69. felt stigmatised^a^	Stigmatisation	0.940	0.936	0.017	0.84413
70. blamed by other people^a^	Stigmatisation	0.937	0.943	0.015	0.69940
71. a finger-wagging from others^a^	Stigmatisation	0.946	0.933	0.019	0.46892
73. been told off by other people^a^	Stigmatisation	0.935	0.931	0.020	0.84845
68. disappointed in oneself^a^	Self-blame	0.949	0.950	0.015	0.92832
72. felt guilty^a^	Self-blame	0.960	0.951	0.015	0.50726
74. angry with oneself^a^	Self-blame	0.950	0.950	0.015	0.99253
Are you currently smoking? If yes, please complete the questions below:	NA	NA	NA	NA	NA
75. thought about quitting smoking	Regretful still smoking	0.839	0.886	0.053	0.37011
76. feel guilty for smoking	Regretful still smoking	0.916	0.879	0.053	0.48273
77. annoyed with oneself for smoking	Regretful still smoking	0.953	0.874	0.054	0.13633
78: disappointed in oneself for smoking	Regretful still smoking	0.902	0.874	0.054	0.60243
79. change in one’s attitude towards smoking	Regretful still smoking	0.805	0.874	0.054	0.19846
80. having second thoughts about one’s smoking	Regretful still smoking	0.856	0.868	0.054	0.82686

^a^for having smoked for many years

### Results of the data collection for the statistical psychometric analyses

158 (63%) men with screening-detected AAA and 275 (55%) men with normal screening results returned the questionnaire. These 433 completed questionnaires were used in all analysis unless otherwise noted.

### Results from the psychometric statistical analyses

#### Part I

##### Dimensionality of part I of the core-questionnaire COS (Table 1)

Three dimensions fitted the partial credit Rasch model forming scales of: ‘anxiety’, ‘sense of dejection’ and ‘negative impact on behaviour’ (Table 2). None of included items possessed DIF.

Three items in the ‘sense of dejection’ scale showed misfit to the partial credit Rasch model and moderate to severe LD was revealed between two pairs of items: items 1&9 and items 1&15. After merging these items into super items in a GLLRM the fit increased substantially (Table 2).

Item 9 in the ‘negative impact on behaviour’ scale showed misfit to the model (Table 3) and at the same time the overall fit to the scale was sufficient, *p* = 0.1761 (Table 2). If item 9 was deleted from the behavioural scale the overall fit of the scale increased and the Cronbach’s alpha did not change.

The four-item dimension ‘negative impact on sleep’ showed misfit where item 23 ‘woken up far too early in the morning’ revealed severe misfit to the model and possessed DIF in relation to social group, income and mother tongue. After deleting item 23 from the sleeping dimension the remaining three items fitted the model, no DIF was identified and only minor LD between items 16 and 24 was revealed.

##### Dimensionality of part I of the AAA-screening-specific items

Except for the three items (65, 66 and 67) in the theme ‘Information’ all the remaining 49 items included in the 13 different new AAA-screening-specific themes fitted their respective Rasch models (Tables 2, 3 and 4). None of these 49 items possessed DIF. In seven of these 13 new scales no LD was revealed among the included items. In the remaining six new scales only minor LD was revealed between some of the items. The three items in ‘Information’ theme did not fit the model and LD was revealed between all three items (Table 2). Item 65 had misfit to the model (Table 3). After deleting item 65, the two remaining items fitted the model and no DIF and no LD were revealed (Tables 2 and 3).

#### Part II

##### Dimensionality of part II of the core-questionnaire COS

In five of six dimensions the items fitted the partial credit Rasch model according to the overall fit statistics (Table 2) and the item fit statistics (Table 5). The six items included in the ‘Existential value’ scale also fitted the Rasch model at item level but revealed marginal misfit at the overall fit statistic’s level. In the ‘Relaxed/calm’ scale item 3 ‘relaxed’ possessed DIF in relation to diagnosis and LD was revealed between item 3 and the two other items. After deleting item 3 from the scale the fit to the model dropped a bit and so did Cronbach’s alpha (Table 2). No DIF was identified the in remaining 20 items in part II of the COS. Moreover, only minor LD was revealed in two item pairs in respectively the ‘Impulsivity’ scale and in the ‘Empathy’ scale.

**Table 5:** Summary of result from the psychometric analyses of part 2 of the COS-AAA

The items of part II of the COS-AAA in order of the scales (the item number indicates the order of appearance in the questionnaire)	Subscales and misfit	Observed	Expected	Gamma sd	Probability of fit to the Rasch model
1. broader aspects of life	Existential values	0.821	0.864	0.031	0.15680
2. enjoyment of life	Existential values	0.913	0.863	0.037	0.17767
8. thought about future	Existential values	0.860	0.860	0.036	0.99225
9. well-being	Existential values	0.886	0.861	0.034	0.46631
10. awareness of life	Existential values	0.840	0.859	0.038	0.62198
11. value life	Existential values	0.852	0.864	0.031	0.70760
3. relaxed	Relaxed/calm	0.842	0.859	0.040	0.65712
7. calm	Relaxed/calm	0.875	0.854	0.036	0.56099
15. relieved	Relaxed/calm	0.844	0.857	0.035	0.70844
4. family	Social relations	0.987	0.986	0.009	0.92118
5. friends	Social relations	0.996	0.984	0.010	0.27472
6. other people	Social relations	0.958	0.984	0.012	0.02807^a^
12. energy	Impulsivity	0.848	0.903	0.030	0.06633
14. lived life to the full	Impulsivity	0.933	0.897	0.039	0.36525
17. being impulsive	Impulsivity	0.913	0.897	0.038	0.68946
19. desire to venture into something new	Impulsivity	0.922	0.905	0.028	0.54384
20. desire to venture into something risky	Impulsivity	0.926	0.910	0.026	0.53982
21. done some things that overstepped one’s bounds	Impulsivity	0.882	0.901	0.034	0.56734
13. responsibility for one’s family	Empathy	0.691	0.752	0.067	0.36721
16. understand other people’s problems	Empathy	0.873	0.889	0.031	0.60584
18. ability to listen to other people’s problems	Empathy	0.896	0.870	0.033	0.42455

^a^Adjusting the *p*-values in the table in order to control the false discovery rate and so avoid spurious significant results due to multiple testing suggested that this result should be regarded as not significant [38]

All the items’ thresholds were in order in each of the Rasch analyses.

## Discussion

In this study, a new condition-specific questionnaire with high content validity and adequate psychometric properties measuring psychosocial consequences of being diagnosed with an asymptomatic AAA has been developed. The core-questionnaire COS that previously has been found to be relevant for participants in breast, lung and cervical cancer screening was also found to be relevant for men offered regular follow-up of an asymptomatic screening-detected AAA. Moreover, 14 new AAA-screening-specific scales were developed and validated encompassing more than 50 new items.

Measuring psychosocial consequences of healthcare interventions is complex and such studies require careful methodological considerations to be able to provide meaningful results. Our study shows that the use of a condition-specific measure in quantitative studies about psychosocial aspects in AAA screening is valid. We had to develop 14 new scales encompassing more than 50 new items in addition to the core questionnaire COS (encompassing 48 core items) before a new questionnaire called the COS-AAA achieved high content validity. This strongly supports that the psychosocial consequences of living with an asymptomatic AAA under surveillance are diverse and multidimensional. Previous quantitative studies on psychosocial consequences of AAA screening have not ensured high content validity or adequate psychometric properties of the questionnaires used [11–16]. Therefore, the findings of our study suggest that the results of these previous studies might not comprehensively and adequately investigate all potential psychosocial consequences of AAA screening.

The incidence of AAA has dropped more than 70% in Sweden [42] and the UK [43] most likely caused by reduced smoking but adjuvant medication for cardiovascular risk factors could also be a plausible explanation. If the incidence of a condition screened for drops, the absolute benefits of a screening programme diminishes, and thereby the benefit-to-harm balance could become less favourable [44]. The drop in incidence of AAA makes it important to adequately measure the potential psychosocial consequences of AAA screening: evidence from longitudinal surveys using a condition-specific measure, e.g. the COS-AAA, are needed to evaluate the benefit-to-harm balance of AAA screening comprehensively and adequately. Another benefit of using a psychosocial condition-specific measure is to identify areas of improvement regarding men undergoing surveillance for AAA. For the present analysis we have collected cross-sectional data from men participating in AAA screening having a normal screening result or being diagnosed with AAA. We are planning in a separate study to publish results on the difference in psychosocial consequences between men diagnosed with AAA and living under surveillance of the aneurysm and men having a normal screening result. Such results could also indicate if the COS-AAA is able to discriminate between these two groups of screening participants. However, because our data are not longitudinal and we do not have two or more repeated measurements, we cannot estimate to what degree and for how long the two groups of men are experiencing psychosocial consequences (e.g. relief or more anxiety) due to their participation in AAA screening. An ideal design for such a study would be to include a baseline measurement before invitation to screening and thereafter follow hundreds of men for years, diagnosed with AAA or having normal screening results.

Item 9 ‘change in appetite’ showed misfit to the Rasch model despite the overall fit of the behavioural scale was sufficient (Tables 2 and 3). This could be a type 1 error or an actual misfit. Before deciding to delete item 9 permanently from the behavioural scale in an AAA setting it would be needed to confirm this misfit to the behavioural scale in additional data collected with the COS-AAA. Item 23 ‘woken up far too early in the morning’ revealed substantial misfit in the sleeping scale both at the item level and at the overall level plus items 23 possessed DIF in relation to three co-variates (Tables 2 and 3). Deleting item 23 from the sleeping scale was followed by a substantial increase in the overall fit statistics indicating that item 23 should be handled as a ‘poor’ item (Table 2). In the sense of dejection scale two pairs of items showed moderate to severe local response dependency (LD). After handling this problem by merging these pairs into so-called super items the fit to the sense of dejection scale improved substantially (Table 2). Therefore, the sense of dejection scale can be used including all six original items. When two or more items in a scale have LD the item information drops. In psychosocial research in healthcare the latent constructs that are wanted to be measured cannot be described in hundreds of nuances, e.g. in how many ways can you ask about sleeping problems without asking the same question? Therefore, if a latent construct in psychosocial healthcare is to be measured, and as many as possible items are generated from interviews to describe different nuances and severities of the latent construct, some degree of redundancy and some degree of LD are inevitable and must be accepted. A result of LD is the drop in item information and thereby theoretically a drop in reliability. This drop in information and reliability can be compensated by using multi-dimensional questionnaires encompassing as many scales as needed to achieve high content validity.

In the new AAA-screening-specific scale ‘Information’ Item 65 ‘missed information about physical activities and aneurysm’ revealed misfit to the Rasch model at the item and overall level (Tables 2 and 3). There was no indication of DIF or LD among the three items in the ‘Information’ scale. Deleting item 65 from the ‘Information’ scale substantially improved the overall fit to the model. Therefore, it cannot be decided if item 65 should be handled as a single item or as a ‘poor’ item would need further psychometric analysis in new datasets collected with COS-AAA.

In part II of the COS the overall fit to the Rasch model for the six items in the ‘Existential values’ scale showed marginal misfit (Table 2). However, all six items revealed sufficient fit statistics at the item level (Table 5). There were no indications of DIF or LD among these six items. Therefore, the most plausible explanation is that the revealed marginal misfit is a type 1 error. Item 3 ‘relaxed’ in the ‘Relaxed/calm’ scale possessed uniform DIF in relation to diagnosis. After deleting item 3 from the scale both the overall fit statistics and Cronbach’s alpha dropped a bit. Therefore, the ‘Relaxed/calm’ scale can be used with all three items as long as the uniform DIF in relation to diagnosis is taken into account [45]. However, a 2-item ‘Relaxed/calm’ scale could also be used.

## Conclusion

A new condition-specific questionnaire, called the COS-AAA (Consequences Of Screening in Abdominal Aortic Aneurysm), with high content validity and adequate psychometric properties measuring psychosocial consequences of being diagnosed with an asymptomatic AAA has been developed. The COS-AAA consists of two parts: part I encompasses 18 scales including more than 70 items and part II encompasses 5 scales including 21 items.

### Implications for practice and research

Our study suggests that results from previous surveys about psychosocial consequences of AAA screening might be of limited value; hence the magnitude and duration of psychosocial harm caused by AAA screening is unknown.

New surveys are needed using the COS-AAA or other condition-specific questionnaires with high content validity and adequate psychometric properties. Such surveys should include a baseline measurement before invitation to screening plus follow-up in a longitudinal design including several measurements in a time period of years.

## Abbreviations

AAA: Abdominal aortic aneurysm
COS: A common core-questionnaire for the three measures above: Consequence of Screening
COS-BC: Consequence of Screening in Breast Cancer
COS-CC: Consequence of Screening in Cervical Cancer
COS-LC: Consequence of Screening in Lung Cancer
DIF: Differential item functioning
GLLRM: Graphical log-linear Rasch models
LD: Local response dependency
QoL: Quality of life
